# Comparative Analysis of the Gut Microbial Communities in Forest and Alpine Musk Deer Using High-Throughput Sequencing

**DOI:** 10.3389/fmicb.2017.00572

**Published:** 2017-04-03

**Authors:** Xiaolong Hu, Gang Liu, Aaron B. A. Shafer, Yuting Wei, Juntong Zhou, Shaobi Lin, Haibin Wu, Mi Zhou, Defu Hu, Shuqiang Liu

**Affiliations:** ^1^Laboratory of Non-invasive Research Technology for Endangered Species, College of Nature Conservation, Beijing Forestry UniversityBeijing, China; ^2^Institute of Wetland Research – Chinese Academy of ForestryBeijing, China; ^3^Forensic Science and Environmental & Life Sciences, Trent University, PeterboroughON, Canada; ^4^Zhangzhou Pien Tze Huang Pharmaceutical Co., LtdZhangzhou, China; ^5^Breeding Centre of Alpine Musk Deer in FengchunLanzhou, China

**Keywords:** gut microbiota, bacterial ecology, symbioses, coevolution, *Moschus berezovskii*, *Moschus chrysogaster*

## Abstract

The gut ecosystem is characterized by dynamic and reciprocal interactions between the host and bacteria. Although characterizing microbiota for herbivores has become recognized as important tool for gauging species health, no study to date has investigated the bacterial communities and evaluated the age-related bacterial dynamics of musk deer. Moreover, gastrointestinal diseases have been hypothesized to be a limiting factor of population growth in captive musk deer. Here, high-throughput sequencing of the bacterial 16S rRNA gene was used to profile the fecal bacterial communities in juvenile and adult alpine and forest musk deer. The two musk deer species harbored similar bacterial communities at the phylum level, whereas the key genera for the two species were distinct. The bacterial communities were dominated by Firmicutes and Bacteroidetes, with the bacterial diversity being higher in forest musk deer. The Firmicutes to Bacteroidetes ratio also increased from juvenile to adult, while the bacterial diversity, within-group and between-group similarity, all increased with age. This work serves as the first sequence-based analysis of variation in bacterial communities within and between musk deer species, and demonstrates how the gut microbial community dynamics vary among closely related species and shift with age. As gastrointestinal diseases have been observed in captive populations, this study provides valuable data that might benefit captive management and future reintroduction programs.

## Introduction

Gut microbiota are an integral component of their host. Microbiota play a key role in host fitness, including the proliferation of enterocytes, the defense against pathogens, the production of secondary metabolites, and the digestion of complex carbohydrates (Flint et al., 2008; Walter et al., 2011). Gut bacteria also harbor opportunistic pathogens, suggesting the gastrointestinal tract is a potential pathway for pathogen invasion (Roeselers et al., 2011). This is especially true for ruminants, where their unique digestive characteristics and microbiome have facilitated adapting to food with a high fiber content, but also made them susceptible to multiple diseases and disorders (Russell and Rychlik, 2001). Accordingly, gut microbiota in ruminants play a more prominent role in the species biology compared to most other animals (Chaucheyras-Durand and Durand, 2010; Fraune and Bosch, 2010; Rosenberg et al., 2010).

Sampling the rumen is not always reflective of the entire microbiome, as different gastrointestinal sections harbor different microbiota (Savage, 1977). In contrast, fecal microbial data represent a combination of gut microbial communities distributed throughout the intestinal tract (Eckburg et al., 2005). Fecal sampling is also non-invasive and is therefore beneficial for endangered or cryptic species. High-throughput sequencing of fecal DNA can elucidate bacterial communities and is attractive because it effectively deals with mixed DNA templates (Hamady et al., 2008) and several recent studies have employed these technologies to explore the microbiota of humans (Yatsunenko et al., 2012), Giant Panda (Xue et al., 2015), cattle (Shanks et al., 2011), horse (Shepherd et al., 2012), elk and white tailed deer (Gruninger et al., 2014). The colonization and diversity of gastrointestinal bacterial communities can also be affected by many biotic and abiotic factors, including host age (Claesson et al., 2011; Jami et al., 2013), host species (Shepherd et al., 2012; Gruninger et al., 2014), host stress (Bailey et al., 2010), host diseases (Andersson et al., 2008; Larsen et al., 2010), diet composition (Shanks et al., 2011), drugs exposure (Dethlefsen et al., 2008), geographical location (Linnenbrink et al., 2013; Maurice et al., 2015; Henderson et al., 2016), and environment (Sullam et al., 2012; Gruninger et al., 2014). Similarly, gut bacterial diversity increases significantly when shifting from carnivory to herbivory (Ley et al., 2008). Multiple comparisons of the rumen microbial community from different hosts have revealed that while the composition of microbiota varies with diet and host species, the core microbiome is generally shared among related host species (Henderson et al., 2016). Importantly, assaying microbial community variation within and among populations can provide informative for conservation and management efforts as variation is linked to host health and nutritional state (Dethlefsen et al., 2007; Sekirov et al., 2010; Hooper et al., 2012) and is critical for commercial ungulate production (Alexander and Plaizier, 2016).

Forest musk deer (FMD, *Moschus berezovskii*) and alpine musk deer (AMD, *Moschus chrysogaster*) are ruminants that are distributed throughout the forests and mountains of East Asia, with China being one of the most important areas for the species (Yang et al., 2003). The musk secreted by adult males is a lucrative raw material used in the perfume industry and for traditional Chinese medicine. Steep declines in wild musk deer populations from over-exploitation and habitat destruction led to breeding programs being established in the 1950s with the aim of providing a sustainable musk resource (Yang et al., 2003). However, the population size of captive musk deer has remained small, partly because of gastrointestinal diseases limiting population growth (Yan et al., 2016). In this study, high-throughput sequencing of 16S rRNA gene was undertaken to characterize the gastrointestinal bacterial communities of two related *Moschus* species. We tested the hypothesis that related hosts harbor similar bacterial communities, and explored how microbial diversity shifted among age classes. This work serves as the first sequence-based analysis of variation in bacterial communities within and between musk deer species and is an important contribution to the study of gut microbial dynamics within and among ruminant species.

## Materials and Methods

### Study Sites and Animals

The FMD breeding center (34°11′ N, 106°50′ E) is located in Aba, Sichuan Province, a region of eastern Tibetan Plateau at an altitude of 2,800 m, with the annual average temperature and rainfall of 11.3°C and 634.6 mm, respectively. The AMD breeding center (35°48′ N, 104°04′ E) is located in Lanzhou, Gansu Province, a region of eastern Qilian Mountains at an altitude of 2,300 m, with the annual average temperature and rainfall of 12.1°C and 564.6 mm, respectively. All musk deer were fed with fresh leaves from April to September, and dried leaves from October to March. Leaves were collected from the natural habitat of musk deer. The leaves for FMD mainly included *Usnea diffracta, Swida bretschneideri, Fraxinus chinensis, Acer mono* and *Clematis armandii*, whereas AMD feed consisted of *Spiraea myrtilloides, Lonicera chrysantha, Lonicera ferdinandii, Acer tetramerum*, and *Cerasus tomentosa*. Several types of grain flour were added to maintain the levels of protein and starch necessary for normal fermentation in rumen. The AMD were dewormed bimonthly, while the FMD were dewormed twice annually. Water was provided *ad libitum*.

### Samples Collection

The musk deer were separated at night to allow for feces to be collected from specific individuals. Ten juvenile (1–1.5 years old; JA1–JA10) and 10 adult (2.5–4 years old; AA1–AA10) AMD were sampled at the breeding center. Ten juvenile (JF1–JF10) and 10 adult FMD (AF1–AF10) were sampled from the FMD breeding center. All selected animals appeared healthy and ear tags were used to distinguish each individual. Fecal matter left in all houses was cleaned out every evening between 18:00 and 20:00 h, which allowed for the collection of fresh feces in the morning. All fresh samples were preserved at liquid nitrogen immediately and transported to our laboratory in a mobile refrigerator, then frozen at -80°C within 12 weeks until DNA extraction. This study was carried out in accordance with the recommendations of the Institution of Animal Care and the Ethics Committee of Beijing Forestry University. The protocol was approved by the Ethics Committee of Beijing Forestry University.

### DNA Extraction

The QIAamp DNA Stool Mini Kit (QIAGEN, Hilden, Germany) was used to extract total bacterial DNA according to the manufacturer’s protocol. The integrity of the nucleic acids were determined visually by electrophoresis on a 1.0% agarose gel containing ethidium bromide. The concentration and purity of each DNA extract were determined using a Qubit dsDNA HS Assay Kit (Life Technologies, Carlsbad, CA, USA). The extracted total DNA was preserved at -80°C.

### PCR Amplification and High-Throughput Sequencing

Polymerase chain reaction (PCR) was performed using purified DNA as the template to amplify the fragment of the 16S rRNA gene. The universal bacterial primers 341F (CCCTACACGACGCTCTTCCGATCTG) and 805R (GACTGGAGTTCCTTGGCACCCGAGAATTCCA; Jakobsson et al., 2014), covering the highly variable V3/V4 region, were modified by adding Miseq barcodes (Illumina, San Diego, CA, USA; Supplementary Table S1). The PCR was run in a total reaction volume of 50 μL. Each reaction mixture contained 5 μL 10× PCR buffer, 0.5 μL dNTP (10 mM each), 0.5 μL Taq DNA polymerase (5 U/μL; Thermo Scientific, Waltham, MA, USA), forward and reverse primers (0.5 μL each, 50 μM), 2 μL DNA template and sterile water. In order to improve the binding efficiency of the primers and the template, the two-step PCR were performed as follows: initial denaturing at 94°C for 3 min, followed by 5 cycles of 30 s at 94°C (denaturing), 20 s at 45°C (annealing) and 30 s at 65°C (extension), then 20 cycles of 20 s at 94°C (denaturing), 20 s at 55°C (annealing) and 30 s at 72°C (extension) and final extension for 10 min at 72°C. The PCR products were purified using the SanPrep Gel Extraction Kit (Sangon Biotech, Shanghai, China) according to the protocol of manufacturer. High-throughput sequencing was performed at Sangon Biotech in Shanghai using the MiSeq PE300 Sequencing System (Illumina, San Diego, CA, USA).

### Statistical and Bioinformatics Analysis

We used the software PRINSEQ (Schmieder and Edwards, 2011) to control for sequence quality. Short reads (<200 bp) and low quality phred (average quality score < 20) were discarded. Sequences with longer homopolymers (>8 bp), or any ambiguous base call in the adapter and barcode sequences were deleted from the dataset. The SILVA bacterial database was used to align the resulting sequences (Schloss, 2009). The pre.cluster and chimeras.uchime commands of Mothur (Schloss et al., 2009) were used to detect and remove chimera sequences. Distance matrices were established using the dist.seqs command with the operational taxonomic units (OTUs) defined by using the furthest neighbor clustering algorithm at phylogenetic similarity of 93–97%. The Good’s coverage estimator was used to confirm the completeness of sampling. The rarefaction curves of OTUs, Good’s coverage and other richness and diversity indices of bacterial community (i.e., ACE, Chao1, Shannon and Simpson) were estimated using the Mothur software (Schloss et al., 2009).

Taxonomic classifications were conducted using the online Ribosomal Database Project (RDP) classifier with a confidence threshold of 80% (Wang et al., 2007). The one-sample Kolmogorov–Smirnov (K–S) test was used to test the normality of the data. General linear model (for the normally distributed data) and generalized linear model (for the non-normally distributed data) were used to quantify the effects of host age and host species on the relative abundance of top five phyla. Independent-sample *t*-test (for the normally distributed data) or Mann–Whitney *U*-test (for the non-normally distributed data) were used to compare the data between groups with the same age or the same species. A sequential Holm–Bonferroni correction was used to control Type I error with the analysis conducted in SPSS ver. 20.0 (IBM, Corp., Armonk, NY, USA). The non-metric multi-dimensional scaling (NMDS) based on the Bray–Curtis similarities of OTU composition was applied to rank the bacterial communities, and a one-way analysis of similarity (ANOSIM) was performed to determine the differences among groups (Clarke and Gorley, 2006). Here the Bray–Curtis similarity index was used as a metric of similarity between the bacterial communities based on the abundance of OTUs between samples. A heatmap analysis was conducted to compare the overall bacterial composition associated with the species and age of hosts. Venn diagrams and statistical clustering were used to determine the shared OTUs by all group members that we define as core microbiome. The heatmap figures and Venn diagrams were produced using R^1^, and the cladogram was generated using the online LEfSe project^2^. The raw sequences obtained in this study were available through the NCBI Sequence Read Archive (accession number SRR5196686).

## Results

### Validation of the Dataset

After a series of procedures to rarify the datasets, 19,589 to 60,611 (Mean number = 33,690 ± 9,206) analyzed sequences (Mean length = 406.3 bp) were obtained from each sample. This resulted in 473,139 sequences from the 40 samples (Supplementary Table S1) with Good’s coverage percentage ranging from 69 to 94% (Mean value = 82%, Supplementary Table S2). A total of 155,325 OTUs were obtained at the 97% sequence similarity cut-off levels, with 8,245 ± 2,427 (range: 2,143 to 12,312) as the mean number of OTUs per sample (Supplementary Table S2). The Shannon index rarefaction curves suggested that more sequencing might identify additional OTUs, whereas, the bacterial diversity of each sample appeared to plateau (**Supplementary Figure S1**). The value of Good’s coverage suggested that more than 80% bacterial phylotypes in the present samples were identified in this study. The proportion of unassigned OTUs at genus level varied between 10.41 and 38.77% among samples, accounting for 27.82% of the full dataset (AMD, 29.28%; FMD, 26.36%; **Supplementary Figure S2**). The unclassified mean rates of OTUs at domain and phylum level were 0.098% (0.003–1.103%) and 0.71% (0.26–1.44%), respectively. The Bray–Cutis similarity of gut bacterial communities within sampling group (0.89 ± 0.07) was significantly higher than among groups (0.85 ± 0.07; *U* = 23039, *p* < 0.001; Supplementary Table S3).

### Core Bacterial Communities in Musk Deer Species

**FIGURE 1:**
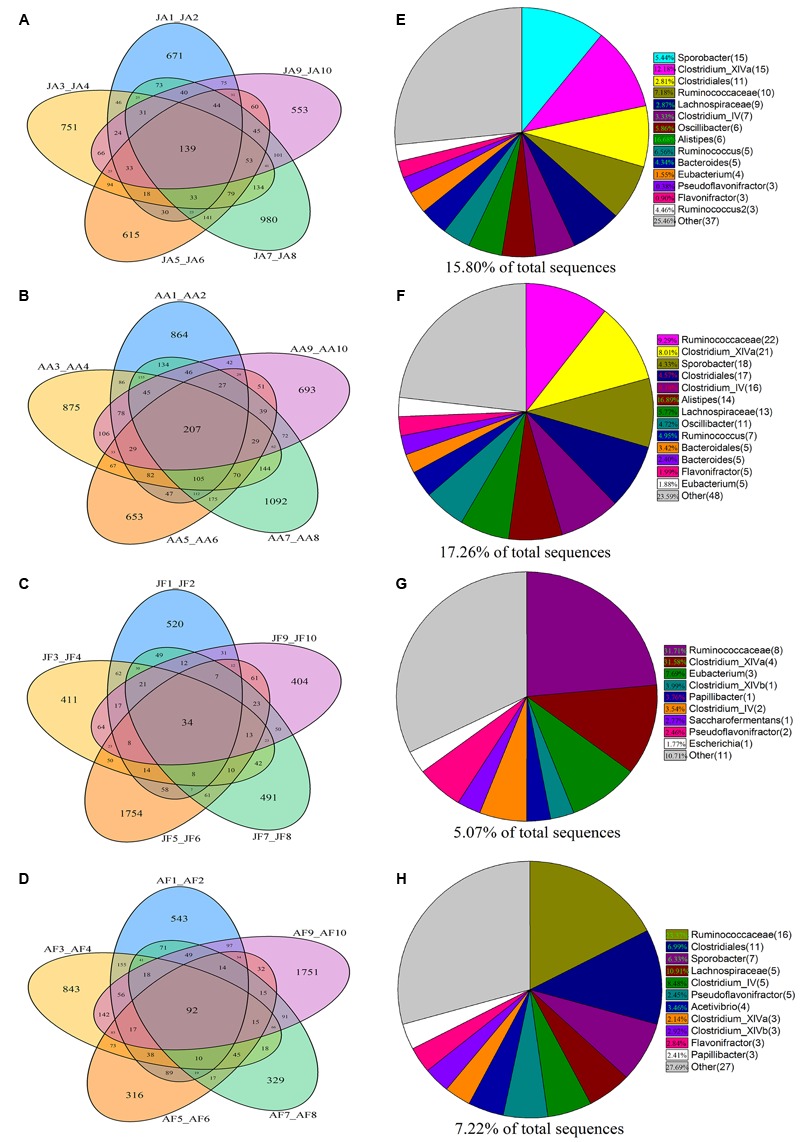
**Distribution of bacterial taxa within the four sample groups.** The Venn diagrams shows the numbers of OTUs (97% sequence identity) that were shared or unshared by the individuals of **(A)** juvenile alpine musk deer (JA), **(B)** adult alpine musk deer (AA), **(C)** juvenile forest musk deer (JF) and **(D)** adult forest musk deer (AF), respectively, depending of overlaps. For presentation two individuals had to be combined (e.g., JA1_JA2) thereby reflecting the number of OTUs shared by those two individuals. The pie diagrams show the core bacterial composition of groups: **(E)** JA, **(F)** AA, **(G)** JF and **(H)** AF. The taxa that occurred at low abundance were included as “other.”

Based on phylogenetic classification, OTUs could be assigned to 45 phyla and 1 unclassified group in the two musk deer species. Here, the shared taxa by all individuals in each group were deemed to be core bacterial communities. We determined the core bacteria for these four sampling groups, and each musk deer group was divided into two age groups. The number of OTUs shared by all individuals within each sampling group was 139, 207, 34, and 92 for the juvenile AMD, adult AMD, juvenile FMD, and adult FMD groups, respectively (**Figures 1A–D**). The core bacterial communities for each sampling group were exhibited in **Figures 1E–H**, and 81.67% of these taxa belonged to Firmicutes (44 taxa) and Bacteroidetes (5 taxa).

### Age-Related Differences in Bacterial Communities between AMD and FMD

**FIGURE 2:**
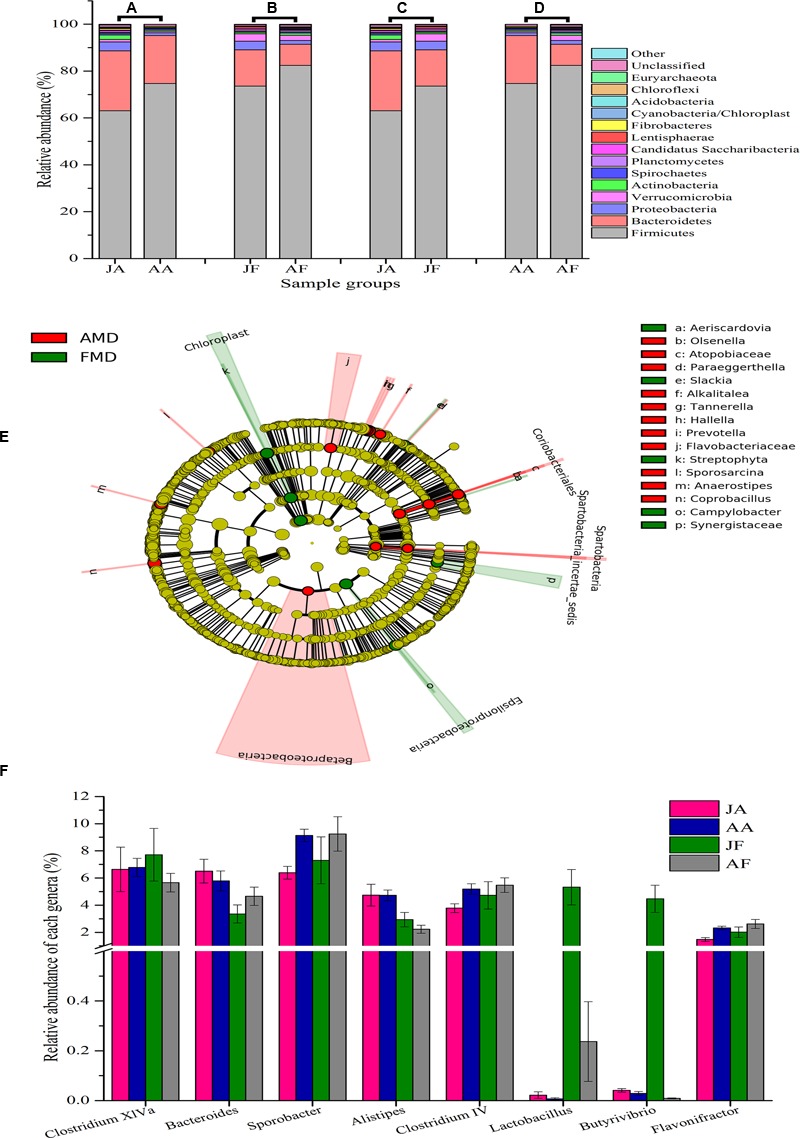
**Bacterial composition of the different sample groups. (A–D)** represent the mean relative abundance of bacterial phyla within each groups. The sequences that could not be classified into any known phyla were assigned as “unclassified,” and the sequences with low mean relative abundance (<0.1%) were assigned as “other.” JA, juvenile alpine musk deer; AA, adult alpine musk deer; JF, juvenile forest musk deer; AF, adult forest musk deer. **(E)** A cladogram showing the differences in relative abundance of taxa at five levels between alpine musk deer (AMD) and forest musk deer (FMD). The plot was generated using the online LEfSe project. The red and green circles mean that AMD and FMD showed differences in relative abundance and yellow circles mean non-significant differences. **(F)** Represents the differences in relative abundance of the top five genera (except the unclassified bacteria) among four sampling groups. The significances were determined using the independent-sample *t*-test.

Gut bacteria showed the most dissimilarity among Firmicutes phyla between juvenile AMD and FMD (**Figure 2C**) and between the adults (**Figure 2D**). Age-related differences in bacterial communities were also observed within the host species (**Figures 2A,B**). Moreover, the cladogram also showed differences in 23 taxa between AMD and FMD (**Figure 2E**). The GLM revealed effects of age and host species on the relative abundance of Firmicutes and Bacteroidetes, whereas the GLMs found no significant effects of age or species on abundance of Proteobacteria, Actinobacteria, and Verrucomicrobia (Supplementary Table S4). The abundance of Firmicutes in FMD was significantly higher than in AMD, whereas the Bacteroidetes in FMD was found markedly lower abundance than in AMD (**Table 1**). Adult musk deer had a higher abundance of Firmicutes than juvenile individuals, while the Bacteroidetes abundance of the adult was lower than the juvenile (**Table 2**). For the Firmicutes to Bacteroidetes ratio, we observed significant differences between juveniles and adults for AMD (2.46 and 4.74; *t* = 2.48, *p* = 0.023) and for FMD (3.65 and 9.12, respectively; *t* = 3.16, *p* = 0.011). At the genus level, *Lactobacillus* (5.32%) and *Butyrivibrio* (4.47%) were the two dominant genera for juvenile FMD, but showed low abundance (<0.1%, **Figure 2F**) in juvenile AMD. The relative abundance differed between AMD juvenile and adult for *Sporobacter* (*t* = 4.14, *p* = 0.001), and *Clostridium* IV (*t* = 2.78, *p* = 0.012), but no significant differences were found in FMD (**Figure 2F**).

**Table 1:** The differences in relative abundance (% ± SD) of five major bacterial phyla between alpine musk deer and forest musk deer.

Major phyla	Juvenile			Adult		
	AMD	FMD		AMD	FMD	
Firmicutes	63.07 ± 4.55	73.58 ± 9.54	*t* = 3.15, *p* = 0.008	74.72 ± 4.43	82.42 ± 3.79	*t* = 4.18, *p* = 0.001
Bacteroidetes	25.60 ± 7.69	15.52 ± 7.92	*t* = 2.89, *p* = 0.01	20.48 ± 4.31	9.01 ± 0.59	*t* = 8.33, *p* < 0.001
Proteobacteria	3.75 ± 5.54	3.66 ± 4.43	*U* = 43.0, *p* = 0.63	1.17 ± 0.63	1.61 ± 0.93	*U* = 39.0, *p* = 0.44
Actinobacteria	1.96 ± 2.04	0.66 ± 0.63	*t* = 1.92, *p* = 0.08	0.54 ± 0.33	0.98 ± 0.84	*t* = 1.56, *p* = 0.14
Verrucomicrobia	1.07 ± 1.15	3.17 ± 3.25	*t* = 1.93, *p* = 0.08	0.70 ± 0.57	2.24 ± 2.28	*t* = 2.08, *p* = 0.06

*The significance of Firmicutes, Bacteroidetes, Actinobacteria, and Verrucomicrobia was determined using the independent-sample *t*-test, whereas the non-parametric Mann–Whitney *U*-test was used to examine the significance of Proteobacteria.*

**Table 2:** The differences in relative abundance (% ± SD) of five major bacterial phyla between juvenile and adult musk deer.

Major phyla	Alpine musk deer			Forest musk deer		
	Juvenile	Adult		Juvenile	Adult	
Firmicutes	63.07 ± 4.55	74.72 ± 4.43	*t* = 5.81, *p* = 0.004	73.58 ± 9.54	82.42 ± 3.79	*t* = 2.72, *p* = 0.019
Bacteroidetes	25.60 ± 7.69	20.48 ± 4.31	*t* = 2.42, *p* = 0.028	15.52 ± 7.92	9.01 ± 0.59	*t* = 2.59, *p* = 0.029
Proteobacteria	3.75 ± 5.54	1.17 ± 0.63	*U* = 38.0, *p* = 0.39	3.66 ± 4.43	1.61 ± 0.93	*U* = 38.0, *p* = 0.36
Actinobacteria	1.96 ± 2.04	0.54 ± 0.33	*t* = 2.18, *p* = 0.06	0.66 ± 0.63	0.98 ± 0.84	*t* = 0.97, *p* = 0.35
Verrucomicrobia	1.07 ± 1.15	0.70 ± 0.57	*t* = 0.93, *p* = 0.37	3.17 ± 3.25	2.24 ± 2.28	*t* = 0.73, *p* = 0.47

*The significance of Firmicutes, Bacteroidetes, Actinobacteria, and Verrucomicrobia was determined using the independent-sample *t*-test, whereas the non-parametric Mann–Whitney *U*-test was used to examine the significance of Proteobacteria.*

**FIGURE 3:**
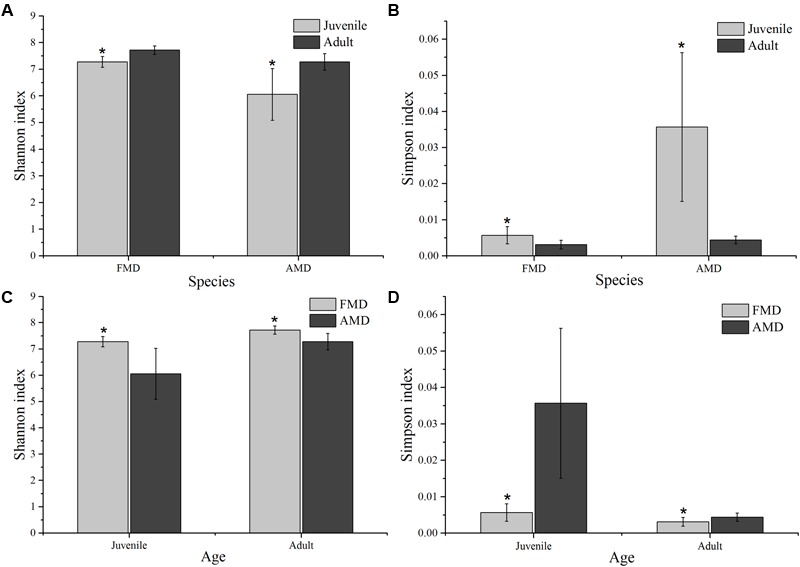
**Age-related differences in microbial diversity (Shannon and Simpson) between alpine (AMD) and forest musk deer (FMD).** Graphs **(A,B)** represent the comparison of Shannon and Simpson index between juvenile and adult. Graphs **(C,D)** represent the comparison of Shannon and Simpson index between AMD and FMD. The significance of Shannon and Simpson indices were determined using the independent-sample *t*-test. ^∗^Means that a significant difference was found.

**FIGURE 4:**
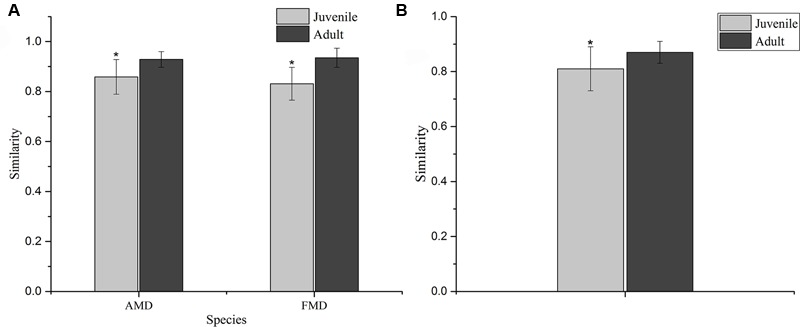
**Similarity of the bacterial communities within (A)** and between **(B)** age groups of alpine (AMD) and forest musk deer (FMD). The similarity was calculated as the average of the pairwise similarity between each paired sample using the Bray–Curtis metric. Y-axis represents the degree of similarity: the closer the similarity is to 1, the higher the average similarity within a group. ^∗^ Represents the significant differences in within-group and between-group similarity between age groups, the significance values have been calculated using *t*-test analysis.

The Shannon index of FMD was significantly higher than AMD (juvenile, *t* = 3.88, *p* = 0.003; adult, *t* = 3.99, *p* = 0.001; **Figure 3C**), and was higher for adult animals compared to juveniles (AMD, *t* = 3.79, *p* = 0.003; FMD, *t* = 5.46, *p* < 0.001; **Figure 3A**). The Simpson index for FMD was significantly lower than AMD (juvenile, *t* = 2.40, *p* = 0.038; adult, *t* = 2.56, *p* = 0.020; **Figure 3D**), as well for adult animals compared to juveniles (AMD, *t* = 2.80, *p* = 0.020; FMD, *t* = 3.06, *p* = 0.009; **Figure 3B**). The average within-group (AMD, *t* = 6.17, *p* < 0.001; FMD, *t* = 9.17, *p* < 0.001) and between-group (*t* = 6.59, *p* < 0.001) similarity analysis showed a significant difference between the age groups, that increased in an age-dependent manner (**Figure 4** and Supplementary Table S3).

**FIGURE 5:**
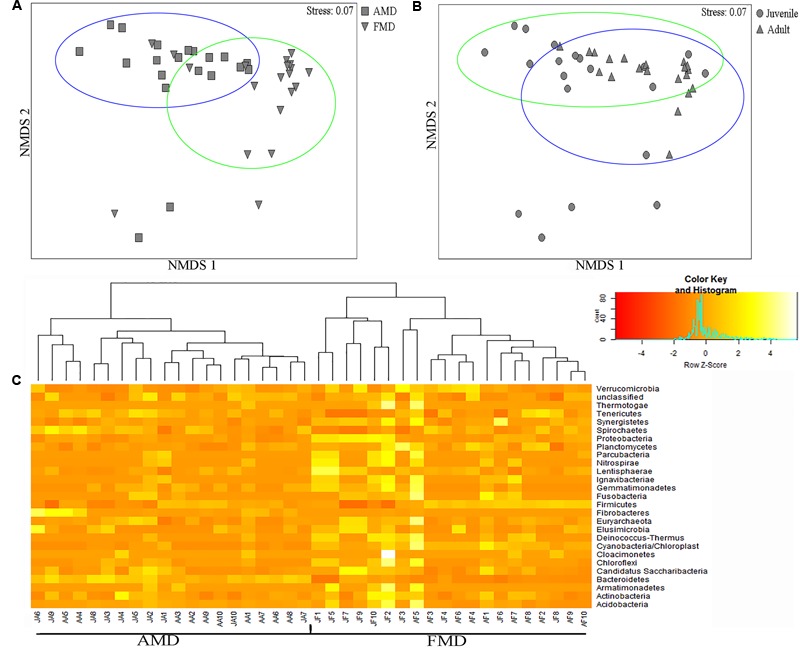
**Non-metric multidimensional scaling (NMDS) and Heatmap analysis of distance between different sample groups. (A,B)** Represent NMDS plots, and the distance between the samples, based on dissimilarity in OTU composition of each sample was calculated using the Bray–Curtis dissimilarity index. Each point represents a different sample and a greater distance between two points infers a higher dissimilarity between them. **(C)** Heatmap analysis of the bacterial distribution among the 40 samples based on hierarchical clustering (Bray–Curtis distance metric and complete clustering method). Each row represents a dominant bacterial phyla where columns represent the 40 individual samples. The values in the Heatmap represent the square root-transformed relative percentage of each bacterial phyla.

The ANOSIM analysis revealed differences in bacterial communities between host species (*R* = 0.52, *p* = 0.001) and age groups (*R* = 0.31, *p* = 0.002), although the NMDS plots showed some overlap among individuals (**Figures 5A,B**). Pairwise ANOSIM indicated documented differences in bacterial communities between two musk deer species (juvenile, *R* = 0.38, *p* = 0.002; adult, *R* = 0.76, *p* = 0.001), which was supported by the NMDS ranking (juvenile, **Supplementary Figure S3C**; adult, **Supplementary Figure S3D**). The pairwise ANOSIM analysis also detected different bacterial communities between juvenile and adult (AMD, *R* = 0.39, *p* = 0.001; FMD, *R* = 0.23, *p* = 0.002), and the NMDS ranking showed dissimilarities between juvenile and adult (AMD, **Supplementary Figure S3A**; FMD, **Supplementary Figure S3B**). The hierarchically clustered heatmap based on the bacterial composition at the phyla level revealed that the bacterial communities in AMD and FMD could be clustered together, whereas it did not show strong clustering of samples by age group (**Figure 5C**).

## Discussion

Our results showed different abundance of microbiotic communities at phylum level between musk deer species. We found that the bacterial diversity and within group similarity increased with age, and this finding indicates that the musk deer gut environment developed into a more restricted niche within the host as the animal aged. The core bacterial phyla of the two musk deer species belonged to Firmicutes and Bacteroidetes (**Figures 2A–D**), which is consistent with previous observations in ruminants (Sundset et al., 2007; Kong et al., 2010; Pandya et al., 2010; Samsudin et al., 2011; Gruninger et al., 2014; Ishaq and Wright, 2014). These phyla dominate the bacterial community of many terrestrial vertebrates suggesting an ecological and functional importance of this group within the gut (Shanks et al., 2011; Li et al., 2016). Firmicutes are the predominant cellulolytic bacteria and degrade fiber into volatile fatty acids for utilization by hosts, while the main function of Bacteroidetes is to degrade carbohydrates and proteins, and facilitate the development of gastrointestinal immune system (Fernando et al., 2010; Jami et al., 2014; Waite and Taylor, 2014; Nuriel-Ohayon et al., 2016). As many phyla could not been classified into further taxa, the differences in relative abundance provides us an overall evaluation of differences in bacterial communities as each phyla typically differs in function (i.e., digestion of fiber, carbohydrate, proteins).

Diet also plays a key role in shaping gut bacterial communities (Li et al., 2012; Scott et al., 2013), with the gastrointestinal tract of ruminants suited for the fermentation of starch and sugars from fibrous plant materials. Importantly, ruminants themselves cannot produce the required fiber-degrading enzymes, which must be generated by colonized bacteria. Thus, the fiber and starch sources in a diet can affect the digestive physiology and ruminal pH, and ultimately result in a specific fecal bacterial community (Zebeli et al., 2008; Belanche et al., 2012). In musk deer, Bacteroidetes were more abundant when individuals were fed a summer diet with more starch, protein, and lactate, whereas, Firmicutes are generally more prevalent in winter diets with more fiber (Fernando et al., 2010). The variation in bacterial phyla observed in musk deer likely reflects the change in the quality and consistency of seasonal diets, as high-starch diets favor more Bacteroidetes with high-fiber diets favoring Firmicutes (Li et al., 2013). Generally, exposure to a high-fiber diet can enrich the gut bacterial diversity (De Filippo et al., 2010; Pitta et al., 2010), but the use of antibiotics can also affect the host–microbe interactions (Russell and Rychlik, 2001; Sullivan et al., 2001). In our system, AMD were dewormed bimonthly, while the FMD were dewormed twice annually. Although the bacterial communities return to pretreatment conditions within several days or weeks after cessation of antibiotic treatment (Kajikawa et al., 2000; Dethlefsen et al., 2008), the bacterial diversity can remain altered (Greenwood et al., 2014); we hypothesize this treatment explains some of the observed differences in musk deer, and combined with local environmental variation likely contributed to the variance among groups.

The diversity (**Figures 3A,B**), within-group similarity and between-group similarity (**Figure 4**) in bacterial communities both increased with age, and is consistent with several previous studies on humans (Palmer et al., 2007; Koenig et al., 2011) and ruminants (Jami et al., 2013). Although the mature gut environment showed a higher diversity of microbial species, it is a more restricted niche with more homogenized bacterial communities. The establishment of the gut bacterial community has been shown to be a progressive process with an increasing diversity and changing composition, which is necessary for development and health of hosts (Jami et al., 2013). Another important aspect of microbial communities is the Firmicutes to Bacteroidetes ratio, as it has been shown to be of relevance in signaling relationship between gut microbial status and aging (Ley et al., 2006; Grilli et al., 2016). Different bacteria must evolve the appropriate strategies and physiological traits to successfully occupy a niche that remains stable during the development of a host (Jami et al., 2013). Juvenile musk deer are undergoing rapid growth, where the adults have a fully developed digestive physiology. More Bacteroidetes colonizing the gut of juveniles could be the result of a deterministic niche, owing to the functional capacity of Bacteroidetes in younger animals. We should note that overall the bacterial communities could be clustered more clearly by species than by host age (**Figure 5**), which could be explained by juveniles attaining adult-like gut microbial composition within 1.5 years of birth, or a general lack of a microbiome-wide signature of age (e.g., Palmer et al., 2007; Benson et al., 2010).

At the genus level, the core genera between these two musk deer species were distinct, and different from the previous study in several ruminant species (Henderson et al., 2016). The abundance of *Lactobacillus* and *Butyrivibrio* in FMD were significantly higher than in AMD (**Figure 2F**), with *Lactobacillus* regarded as an indicator of a healthy bacterial community because it plays an important role in the microecological balance of the host (Floch, 2011; Inglin et al., 2015). *Sporobacter* and *Clostridium* IV were significant higher in adults, while *Bacteroides* abundance decreased from juvenile to adult. Several studies have reported a similar pattern where many protective commensal bacteria, such as *Bacteroides* and *Bifidobacteria*, showed a reduction with age (Woodmansey, 2007; Rey et al., 2014; Arboleya et al., 2016). Interestingly, an average of 27.82% of sequences could not been classified into any unknown genera, suggesting that most likely represent novel bacteria. This discovery is consistent with vast identification of novel species in the gastrointestinal bacteria of wild (Gruninger et al., 2014) and domesticated (Shepherd et al., 2012) ruminants, suggesting that the gut of musk deer harbors a larger bacterial diversity than previously recognized. A detailed phylogenetic characterization of unclassified sequences and their phylogeny will be important future research.

Animal health is inevitably related to the stability of its relevant microbial communities. Thoroughly understanding microbial communities via high-throughput sequencing allows for more robust assessments as to how environmental factors and biological processes shaping the composition and dynamics of the microbial communities; this is an important component of the management and production of ungulates. The present study showed that musk deer species harbored distinct gut bacterial communities, which also altered with aging of the host. This information should be informative for current conservation and management practice, for example altered diet or antibiotic regimes, and future reintroduction programs as gastrointestinal disease appear to be a limiting factor in captive populations (Yan et al., 2016). An important next step will be trying to link microbial diversity to individual musk deer health and characterizing the novel OTUs. Overall, this study provides the first quantification of the musk deer gut microbial community and the factors driving variation that will be useful for understanding the gastrointestinal disorders impacting captive populations.

## Author Contributions

XH and GL carried out the sample collection, DNA extraction, data analysis and drafted the manuscript. AS participated in drafting the manuscript and analysis. YW and JZ participated in the sample collection and DNA extraction. SBL participated in the sample collection and data analysis. HW and MZ participated in the sample collection. DH and SL, who are the corresponding authors, conceived of the study and participated in its design and coordination and helped to draft the manuscript. All authors read and approved the final manuscript.

## Conflict of Interest Statement

The authors declare that the research was conducted in the absence of any commercial or financial relationships that could be construed as a potential conflict of interest.

## Footnotes

1 http://www.r-project.org/

2 https://huttenhower.sph.harvard.edu/galaxy/

## Supplementary Material

FIGURE S1 **The rarefaction curves of OTUs and Shannon index for the 40 samples.** JA1–JA10 represent the samples collected from the juvenile alpine musk deer, AA1–AA10 represent the samples collected from the adult alpine musk deer, JF1–JF10 represent the samples collected from the juvenile forest musk deer, AF1–AF10 represent the samples collected from the adult forest musk deer.

FIGURE S2 **The operational taxonomic unit (OTU) numbers of classified and unclassified bacterial phylotypes among four sample groups.** JA, juvenile alpine musk deer; AA, adult alpine musk deer; JF, juvenile forest musk deer; AF, adult forest musk deer.

FIGURE S3 **Pairwise non-metric multidimensional scaling (NMDS) of the dissimilarity between different sample groups.** Distance between the samples, based on dissimilarity in OTU composition of each sample was calculated using the Bray–Curtis dissimilarity index. Each point represents a different sample and a greater distance between two points infers a higher dissimilarity between them. **(A)** Represents the differences between JA and AA groups; **(B)** represents the differences between JF and AF groups; **(C)** represents the differences between JA and JF groups; **(D)** represents the differences between AA and AF groups. JA, juvenile alpine musk deer; AA, adult alpine musk deer; JF, juvenile forest musk deer; AF, adult forest musk deer.
