# Supplementary figures and images for: Comparative Analysis of the Gut Microbial Communities in Forest and Alpine Musk Deer Using High-Throughput Sequencing
Source: Front Microbiol. 2017 Apr 3;8:572. doi: 10.3389/fmicb.2017.00572 (PMC5376572; doi:10.3389/fmicb.2017.00572)

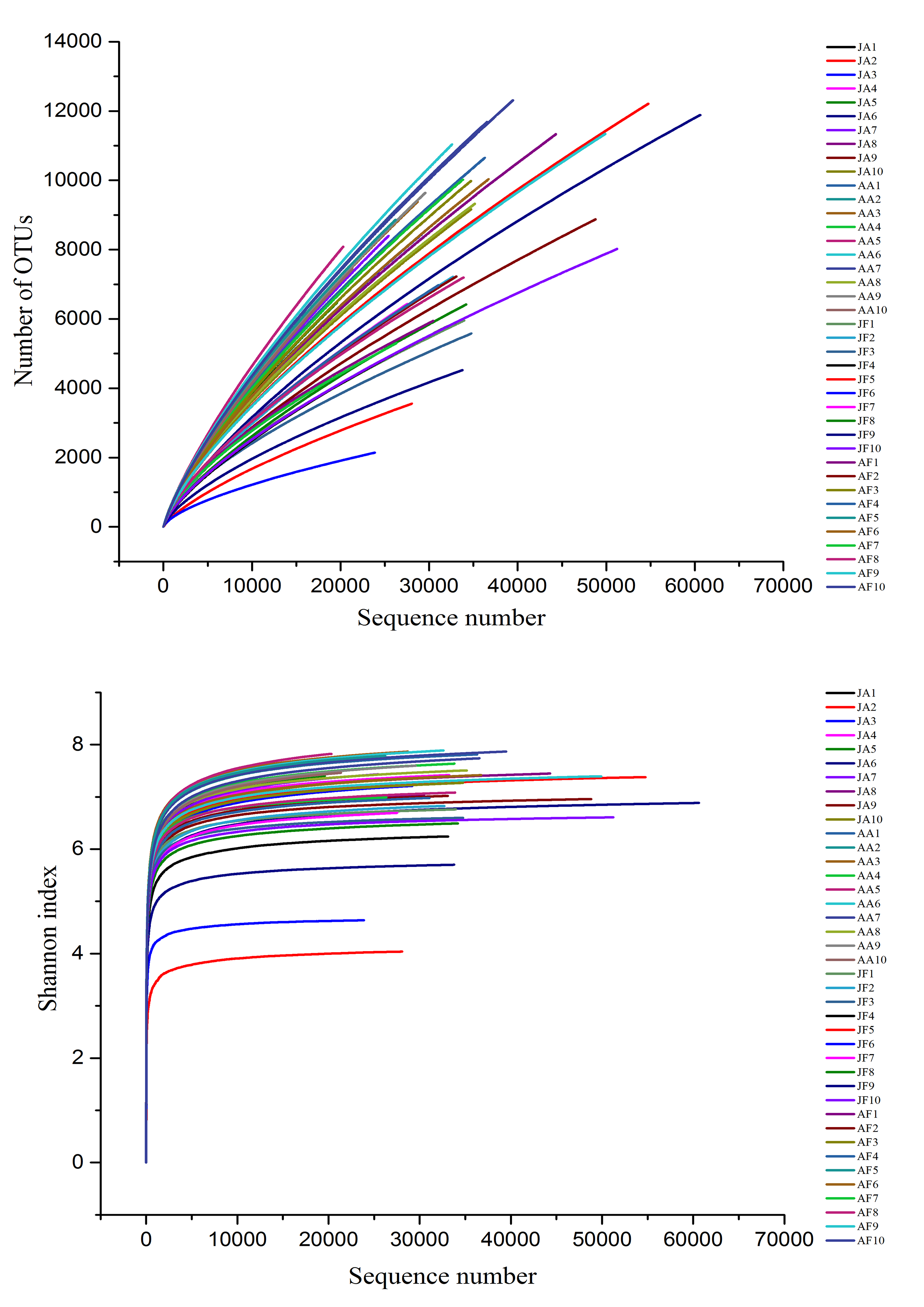

Supplement: FIGURE S1 — The rarefaction curves of OTUs and Shannon index for the 40 samples. JA1–JA10 represent the samples collected from the juvenile alpine musk deer, AA1–AA10 represent the samples collected from the adult alpine musk deer, JF1–JF10 represent the samples collected from the juvenile forest musk deer, AF1–AF10 represent the samples collected from the adult forest musk deer. [file Image_1.TIF]

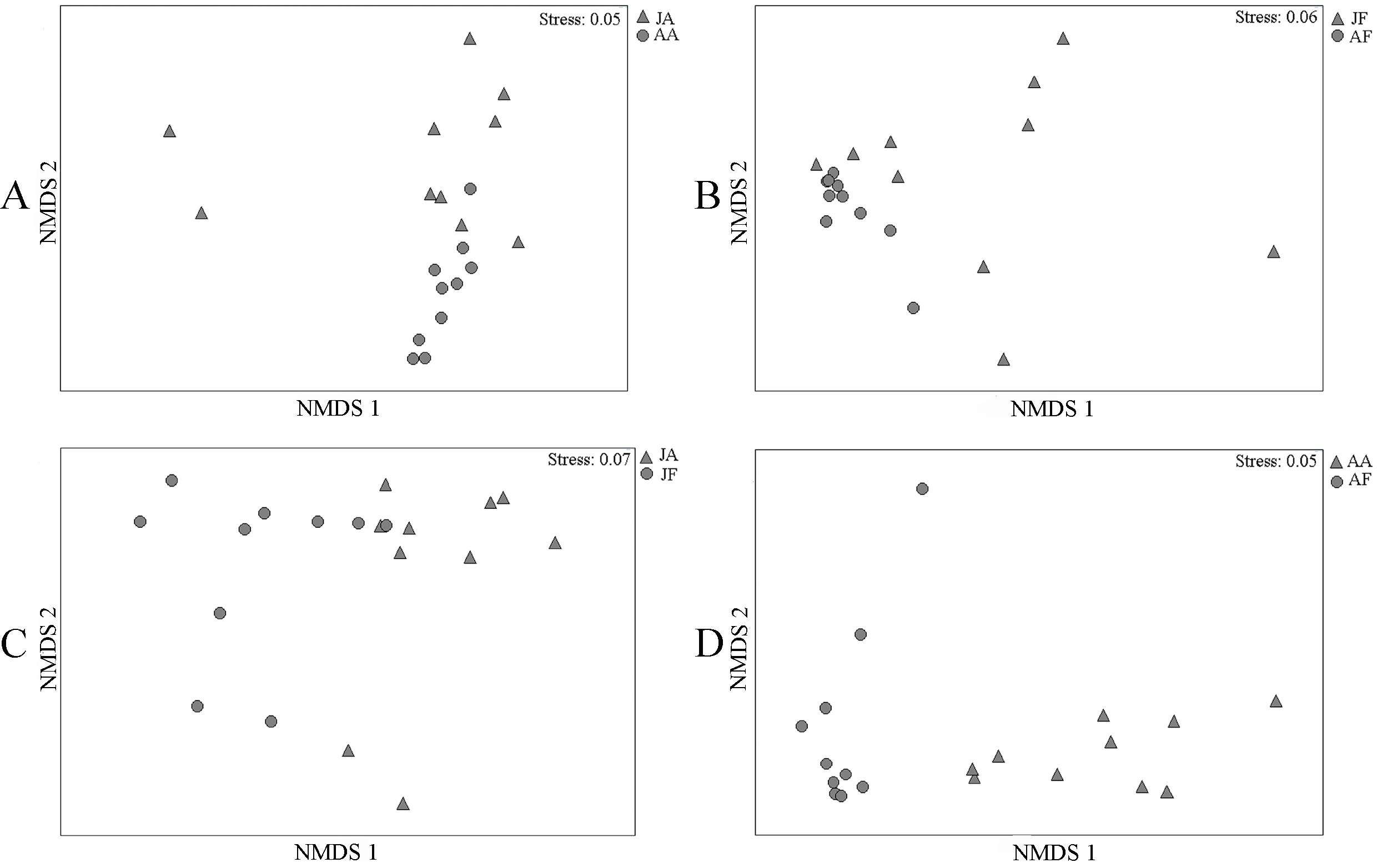

Supplement: FIGURE S3 — Pairwise non-metric multidimensional scaling (NMDS) of the dissimilarity between different sample groups. Distance between the samples, based on dissimilarity in OTU composition of each sample was calculated using the Bray–Curtis dissimilarity index. Each point represents a different sample and a greater distance between two points infers a higher dissimilarity between them. (A) Represents the differences between JA and AA groups; (B) represents the differences between JF and AF groups; (C) represents the differences between JA and JF groups; (D) represents the differences between AA and AF groups. JA, juvenile alpine musk deer; AA, adult alpine musk deer; JF, juvenile forest musk deer; AF, adult forest musk deer. [file Image_3.TIF]
