# Supplementary material for: Comparative Analysis of the Gut Microbial Communities in Forest and Alpine Musk Deer Using High-Throughput Sequencing
Source: Front Microbiol. 2017 Apr 3;8:572. doi: 10.3389/fmicb.2017.00572 (PMC5376572; doi:10.3389/fmicb.2017.00572)
Supplement: Supplementary file 4 [file Table_1.DOC]

**Table S1**

List of the barcodes used to tag each PCR product analyzed in the study, and the effective sequence number and length for each sample. JA1-JA10 represent the samples collected from the juvenile alpine musk deer, AA1-AA10 represent the samples collected from the adult alpine musk deer, JF1-JF10 represent the samples collected from the juvenile forest musk deer, AF1-AF10 represent the samples collected from the adult forest musk deer.

| Samples | Barcodes | Sequence number | Sequence length |
| --- | --- | --- | --- |
| JA1 | AGAACA | 21231 | 412.0 |
| JA2 | AGAGAC | 54741 | 409.7 |
| JA3 | TATGCA | 29165 | 408.5 |
| JA4 | GTAACA | 33227 | 406.1 |
| JA5 | GCGAGG | 19589 | 407.2 |
| JA6 | CGGATG | 60611 | 407.3 |
| JA7 | GTGAAA | 25435 | 407.7 |
| JA8 | ATCTTG | 44309 | 409.3 |
| JA9 | ACTGCG | 48787 | 410.5 |
| JA10 | GTATCT | 34742 | 412.6 |
| AA1 | GGTGTG | 36283 | 406.5 |
| AA2 | AAGGTA | 26600 | 406.1 |
| AA3 | ATCACG | 28686 | 406.2 |
| AA4 | TATCTG | 33814 | 407.5 |
| AA5 | GTTGTT | 20297 | 407.3 |
| AA6 | CGTGGT | 32588 | 406.7 |
| AA7 | CCTTCT | 39465 | 407.2 |
| AA8 | TTGTAG | 35161 | 408.1 |
| AA9 | AACTAT | 29558 | 405.4 |
| AA10 | TTAATT | 21342 | 405.7 |
| JF1 | CGATGT | 33947 | 408.1 |
| JF2 | CGGTTA | 32692 | 405.6 |
| JF3 | AGGAAC | 34754 | 406.0 |
| JF4 | AGAGTA | 33097 | 405.2 |
| JF5 | CGGACC | 28055 | 418.7 |
| JF6 | CTTCAG | 23870 | 356.1 |
| JF7 | TGCATC | 27544 | 408.2 |
| JF8 | TCAGTA | 34173 | 404.6 |
| JF9 | CGGCAC | 33768 | 406.7 |
| JF10 | AGACTG | 51219 | 407.2 |
| AF1 | TCCTGT | 30451 | 407.6 |
| AF2 | GAAGGC | 33060 | 407.0 |
| AF3 | ATGTCA | 34716 | 406.2 |
| AF4 | TTGCTC | 31062 | 405.6 |
| AF5 | TGCTTA | 26211 | 412.9 |
| AF6 | AGTGGC | 36720 | 405.4 |
| AF7 | TATTCT | 26330 | 405.3 |
| AF8 | TACCAC | 33878 | 409.3 |
| AF9 | GAGATA | 49893 | 407.0 |
| AF10 | TTCCGG | 36556 | 401.6 |
