# Supplementary material for: Comparative Analysis of the Gut Microbial Communities in Forest and Alpine Musk Deer Using High-Throughput Sequencing
Source: Front Microbiol. 2017 Apr 3;8:572. doi: 10.3389/fmicb.2017.00572 (PMC5376572; doi:10.3389/fmicb.2017.00572)
Supplement: Supplementary file 5 [file Table_2.DOC]

**Table S2**

**Number of OTUs, estimated OTU richness (ACE and Chao1), diversity index (Shannon and Simpson) and estimated sample Coverage for the different samples. JA, juvenile alpine musk deer; AA, adult alpine musk deer; JF, juvenile forest musk deer; AF, adult forest musk deer.**

| Samples | | OTUs | | ACE | | Chao1 | | Shannon | | Simpson | | Coverage (%) | |
| --- | --- | --- | --- | --- | --- | --- | --- | --- | --- | --- | --- | --- | --- |
| JA1 | 6644 | | 45978 | | 24000 | | 6.76 | | 0.005 | | 77.11 | |  |
| JA2 | 12210 | | 83174 | | 41651 | | 6.83 | | 0.008 | | 83.87 | |  |
| JA3 | 8443 | | 61826 | | 30535 | | 6.60 | | 0.007 | | 78.44 | |  |
| JA4 | 8938 | | 63780 | | 32443 | | 6.24 | | 0.016 | | 80.18 | |  |
| JA5 | 6472 | | 47646 | | 23560 | | 4.04 | | 0.217 | | 75.26 | |  |
| JA6 | 11886 | | 70734 | | 37891 | | 4.64 | | 0.052 | | 86.06 | |  |
| JA7 | 8400 | | 67889 | | 31149 | | 6.69 | | 0.012 | | 74.85 | |  |
| JA8 | 11331 | | 79170 | | 39486 | | 6.49 | | 0.009 | | 81.19 | |  |
| JA9 | 8873 | | 58788 | | 28912 | | 5.70 | | 0.024 | | 87.07 | |  |
| JA10 | 9167 | | 72204 | | 32806 | | 6.61 | | 0.007 | | 80.50 | |  |
| AA1 | 10648 | | 83278 | | 39226 | | 7.03 | | 0.004 | | 78.20 | |  |
| AA2 | 9241 | | 72775 | | 34045 | | 7.02 | | 0.005 | | 73.79 | |  |
| AA3 | 9380 | | 77665 | | 33716 | | 7.40 | | 0.005 | | 75.60 | |  |
| AA4 | 10021 | | 78744 | | 36033 | | 6.98 | | 0.006 | | 77.92 | |  |
| AA5 | 8083 | | 69640 | | 31060 | | 7.79 | | 0.002 | | 69.24 | |  |
| AA6 | 11036 | | 86802 | | 41995 | | 7.42 | | 0.005 | | 74.36 | |  |
| AA7 | 12312 | | 87466 | | 43107 | | 6.99 | | 0.004 | | 76.83 | |  |
| AA8 | 9323 | | 65086 | | 31389 | | 7.08 | | 0.005 | | 80.74 | |  |
| AA9 | 9640 | | 74044 | | 35131 | | 7.39 | | 0.004 | | 75.36 | |  |
| AA10 | 7441 | | 60865 | | 27654 | | 7.74 | | 0.004 | | 73.57 | |  |
| JF1 | 5956 | | 38037 | | 19396 | | 7.38 | | 0.004 | | 87.83 | |  |
| JF2 | 7215 | | 49559 | | 25019 | | 7.38 | | 0.004 | | 83.79 | |  |
| JF3 | 5578 | | 32537 | | 16534 | | 7.21 | | 0.009 | | 89.19 | |  |
| JF4 | 5910 | | 37545 | | 19204 | | 7.42 | | 0.004 | | 87.31 | |  |
| JF5 | 3555 | | 24994 | | 12475 | | 7.40 | | 0.003 | | 90.78 | |  |
| JF6 | 2143 | | 10419 | | 6028 | | 6.88 | | 0.010 | | 94.15 | |  |
| JF7 | 6431 | | 45874 | | 22259 | | 7.43 | | 0.005 | | 83.07 | |  |
| JF8 | 6417 | | 39952 | | 20094 | | 7.45 | | 0.005 | | 86.59 | |  |
| JF9 | 4523 | | 26549 | | 13851 | | 6.96 | | 0.005 | | 90.86 | |  |
| JF10 | 8021 | | 48883 | | 24963 | | 7.27 | | 0.008 | | 89.05 | |  |
| AF1 | 5943 | | 28539 | | 15915 | | 7.81 | | 0.002 | | 87.30 | |  |
| AF2 | 7223 | | 48359 | | 25041 | | 7.76 | | 0.003 | | 84.41 | |  |
| AF3 | 9981 | | 71059 | | 34986 | | 7.87 | | 0.002 | | 78.72 | |  |
| AF4 | 6974 | | 47201 | | 23129 | | 7.64 | | 0.003 | | 83.91 | |  |
| AF5 | 8860 | | 65624 | | 32112 | | 7.82 | | 0.002 | | 74.88 | |  |
| AF6 | 10034 | | 69740 | | 34191 | | 7.89 | | 0.002 | | 79.92 | |  |
| AF7 | 5305 | | 28733 | | 16141 | | 7.87 | | 0.003 | | 86.50 | |  |
| AF8 | 7197 | | 45879 | | 23075 | | 7.51 | | 0.004 | | 85.17 | |  |
| AF9 | 11340 | | 81774 | | 39578 | | 7.60 | | 0.005 | | 83.43 | |  |
| AF10 | 11688 | | 78757 | | 38470 | | 7.46 | | 0.005 | | 76.40 | |  |
