# Supplementary material for: Comparative Analysis of the Gut Microbial Communities in Forest and Alpine Musk Deer Using High-Throughput Sequencing
Source: Front Microbiol. 2017 Apr 3;8:572. doi: 10.3389/fmicb.2017.00572 (PMC5376572; doi:10.3389/fmicb.2017.00572)
Supplement: Supplementary file 6 [file Table_3.DOC]

**Table S3**

The within and among sampling group Bray–Curtis similarity. JA, juvenile alpine musk deer; AA, adult alpine musk deer; JF, juvenile forest musk deer; AF, adult forest musk deer.

| Within or among group | Bray–Curtis similarity (Mean ± SD) |
| --- | --- |
| AA | 92.88 ± 3.18 |
| AF | 93.48 ± 3.85 |
| JA | 85.87 ± 6.91 |
| JF | 83.17 ± 6.51 |
| AA-AF | 86.66 ± 4.18 |
| JA-JF | 80.92 ± 7.64 |
| JA-AA | 85.16 ± 6.20 |
| JF-AF | 85.45 ± 7.42 |
