# Supplementary material for: Comparative Analysis of the Gut Microbial Communities in Forest and Alpine Musk Deer Using High-Throughput Sequencing
Source: Front Microbiol. 2017 Apr 3;8:572. doi: 10.3389/fmicb.2017.00572 (PMC5376572; doi:10.3389/fmicb.2017.00572)
Supplement: Supplementary file 7 [file Table_4.DOC]

**Table S4**

The ANOVA tests of the effects of age and host species on the relative abundance of five major bacterial phyla. The significances of Firmicutes and Bacteroidetes were determined using the General Linear Model, whereas the Generalized Linear Models was used to examine the significances of Proteobacteria, Actinobacteria and Verrucomicrobia.

| Major phyla | Age | Host species |
| --- | --- | --- |
| Firmicutes | F=28.80, p<0.001 | F=22.76, p<0.001 |
| Bacteroidetes | F=9.62, p=0.004 | F=33.00, p<0.001 |
| Proteobacteria | W=2.60, p=0.32 | W=0.03, p=0.87 |
| Actinobacteria | W=2.54, p=0.11 | W=1.50, p=0.22 |
| Verrucomicrobia | W=1.07, p=0.30 | W=2.50, p=0.40 |
